# Quintuple Alliance

**Published:** 1859-05

**Authors:** 


### QUINTUPLE ALLIANCE.

Sometime ago a stranger came into our office whom we were glad to see, when we found out that he was from the sterling old " North State," to which almost as many " Journals" are sent, as any other in the Union, owing partly to his personal influence; and several letters having passed between us, he was in the light of an old acquaintance. Within a day or two we received the following off-hand note, which we should judge was written on his safe arrival at home, before he had taken time to kiss his wife and children. By this it would seem that another name is added to the small list of editors, who are at this time regarded as entitled to that distinctive appellation, " The." Our readers will have no difficulty in calling up at once four names, as towering above the twenty thousand others in this broad Union of States, and all in Gotham too! as if it were the vast center, where all greatness met. The names alphabetically, are Bennett, Dixon, Greely, Willis; all known wherever the English language is read, as " Peers of the Realm" Periodical; each a head and shoulders taller than any of his class. Bennett for his brave enterprises in throwing a vigor and an energy into newspaper life, which it never knew before; Dixon of the Scalpel, for his persistent and fearless onslaught against sham and show, and hollow pretence in medicine and morals, for the merciless blows, scathing and keen, which he inflicts on the twin brothers, assumption and ignorance; and not least, for that genius, profundity and medical scholarship which turn out whole pages in an hour of what is beautiful in reading, and blessed in practice, to wit, lessons of wisdom, safety and truth in reference to health and disease.

Next comes Greeely, the "philosopher" so called, a man who has made himself; who travelled from the clod to congress; a representative man, standing up always and every where for what he believed to be the right, never fainting, never faltering, the friend of the poor and the oppressed, the lover of his kind. Next comes Willis, and at once there rises before us in imagination, for we have never seen him, the personification of grace, the embodiment of the gentleman, with visions of all that is sweet in poetry; and in prose, sparkling, elegant, and pure. Now "truly," as our dear little Alice says, we are ashamed entirely, to say any thing about the quality and quantity, of the fifth individual, for he ought not to be mentioned in the same day, with that breadth of enterprise which marks the first, with that genius and medical lore which distinguishes the second; with that power and daring against all odds, of the third; nor with the high culture, which has enabled the fourth to make his mark amongst the men of his time. Now really, we wish we had not begun this article, and but for our habit of never going backwards, we certainly would throw it in the waste basket, not for any thing which has been written, but for the "falling off" there must be, in what is to be written, but as old North Carolina has "made the best of it," we will give the letter.

March 19, 1859.

Dear Doctor.

I didn't get sick, although I must confess the inducement was great. The idea of *Dr. Hall coming to visit me!* Yes, sir, your promise was instantly recorded, and whenever your New Yoik February weather seemed to affect me, I remembered your promise, and had no dread of sickness. *That* morning's very brief interview with you gave me infinite satisfaction. The purpose of the call was more than a double one. First, I had a great desire to see the living Dr. Hall. I had been paying my dollar per annum, for several years past, to hear from and know of him, but the real live Doctor, and editor of *the* "Journal of Health" it had never been my privilege to see face to face. Now I have seen the man, whom, were I to describe in a few words, I would say, "The comfortable and satisfied Doctor and gentleman. The practical man, the man who never wants to be idle."

I thank you Doctor for the pleasure you have given me and mine. We all read your good sense, and but one complaint have I heard at our fireside, *i. e.*, after every word you write for the Journal has been read, the complaint is "there is not enough of it." That compliment can rarely be paid to the monthlies of the present day; rather too much than too little.

Upon my return, our mutual friend Dr. Thomas, lost no time in asking after you. "Tell me about Dr. Hall," was the first command after "How are you." I did tell him, and told him all I knew.

Excuse me Doctor for the liberty, but I couldn't help telling you how much you are esteemed and respected by your friend and obedient servant,

---

We presume that mere notoriety is, to all great minds, an annoyance; to such it must be a pleasure and a relief to pass along as one of the undistinguishable crowd; there must be rest and sweetness in that privacy. But he that would have a well spring of pure joy rising to his lips perennially, let him aim for the celebrity which quiet, busy, unpublished well-doing never fails to secure after the work has been done, and the worker has gone to get his reward. Thus, while he escapes the idle or impertinent gaze of the crowd, he can at the same time be drinking the nectar, made of the consciousness of having done good with a pure motive, and a true heart; of having done good, in the love of it; this will be an oil to throw on the troubled waters of "late in life," it will soothe the unescapable griefs of age, and gradually prepare the heart for that more perfect rest, where "sorrow and sighing shall flee away."

